# Plasmonic Emission of Bullseye Nanoemitters on Bi_2_Te_3_ Nanoflakes

**DOI:** 10.3390/ma13071531

**Published:** 2020-03-26

**Authors:** Qigeng Yan, Xiaoli Li, Baolai Liang

**Affiliations:** Hebei Key Laboratory of Optic-Electronic Information and Materials, College of Physics Science & Technology, Hebei University, Baoding 071002, China; xiaolixiaoli1999@126.com

**Keywords:** Bi_2_Te_3_ nanoflakes, bullseye nanostructure, cathodoluminescence, plasmonic resonance

## Abstract

Topological insulators, such as Bi_2_Te_3_, have been confirmed to exhibit plasmon radiation over the entire visible spectral range. Herein, we fabricate bullseye nanoemitters, consisting of a central disk and concentric gratings, on the Bi_2_Te_3_ nanoflake. Due to the existence of edge plasmon modes, Bi_2_Te_3_ bullseye nanostructures are possible to converge light towards the central disk. Taking advantage of the excellent spatial resolution of cathodoluminescence (CL) characterization, it has been observed that plasmonic behaviors depend on the excitation location. A stronger plasmonic intensity and a wider CL spectral linewidth can be obtained at the edge of the central disk. In order to further improve the focusing ability, a cylindrical Pt nanostructure has been deposited on the central disk. Additionally, the finite element simulation indicates that the electric-field enhancement originates from the coupling process between the plasmonic emission from the Bi_2_Te_3_ bullseye and the Pt nanostructure. Finally, we find that enhancement efficiency depends on the thickness of the Pt nanostructure.

## 1. Introduction

Three-dimensional topological insulators (3D TIs) have attracted dramatic interests since they perform as insulating materials in the bulk but have metallic surface states [1]. Among all the 3D TIs, Bi_2_Te_3_ is well investigated as a thermoelectric material [2,3]. Recently, it is reported that Bi_2_Te_3_ nanostructures have the possibility to generate plasmonic resonance [4,5,6]. Traditional plasmonic materials are mainly noble metals, such as Ag and Au [7]. Although these materials have been widely studied due to their strong electric field enhancements, they usually suffer from high resistive loss and low tunability [8,9]. The application of Bi_2_Te_3_ in relative fields is significant to extend the choice of plasmonic materials beyond noble metals. The negative real part of the permittivity (ε_1_^’^) is prerequisite for the existence of surface plasmons. Therefore, the surface plasmonic radiation is available across the visible spectral range for Bi_2_Te_3_ because ε_1_^’^ is negative from 240 to 798 nm [5]. Reported by Toudert et al., results of the plasmonic quality factor indicates that Bi_2_Te_3_ is possible to have a stable plasmonic property across the visible spectral range [10]. Moreover, the research on the figure of merit (FOM) of 3D TIs suggests that the plasmonic property of Bi_2_Te_3_ is better than Au under 570 nm, and better than Ag under 420 nm [11]. Consequently, Bi_2_Te_3_ plasmonic nanostructures are expected to achieve numerous applications, including plasmonic lenses [12], biosensors [13], and nanoemitters [14]. 

The enhanced plasmonic emission caused by the localized charge oscillation in Bi_2_Te_3_ nanoflakes can be excited by both laser and electron beam [4,5,6]. Due to the high excitation energy, multiple surface plasmon modes of hexagonal Bi_2_Te_3_ nanoplates were investigated by electron energy loss spectroscopy (EELS) [4]. Observed by photoemission electron microscopy (PEEM), edge plasmon modes were found to be dominant [5]. In order to improve the optical performance of Bi_2_Te_3_ nanostructures, it is critical to have better utilization of these plasmon modes. Structures with periodic gratings, such as bullseye structures, are appropriate candidates to generate enhanced electric field on the Bi_2_Te_3_ [15].

In this report, we propose a Bi_2_Te_3_ bullseye nanoemitter with an emission-enhancement unit in the center. Due to the confinement of concentric gratings, the Bi_2_Te_3_ bullseye nanoemitter is expected to provide an outstanding optical focusing property [12]. It is also reported that an extra plasmonic nanostructure located at the center of the bullseye could efficiently improve the field enhancement [16,17]. Consequently, we directly exfoliate Bi_2_Te_3_ flakes from a bulk crystal and modify the size and geometry by focused ion beam (FIB) milling [18]. Then, a Pt nanostructure is grown by the electron-beam assisted deposition of the gas phase precursor. Bi_2_Te_3_ nanostructures are excited by a high energy electron beam to activate the plasmonic emission. Taking advantage of the spatial resolution of the electron beam, we find that the plasmonic behavior of the Bi_2_Te_3_ bullseye nanoemitter depends on the excitation position and the Pt thickness. The milling and characterization procedures are acquired in the same chamber to avoid the optical loss due to the absorbance of carbon and oxygen [19]. Additionally, the finite element method has been applied to reveal the electric field distribution at the peak wavelength.

## 2. Materials and Methods 

### 2.1. Materials

**Figure 1:**
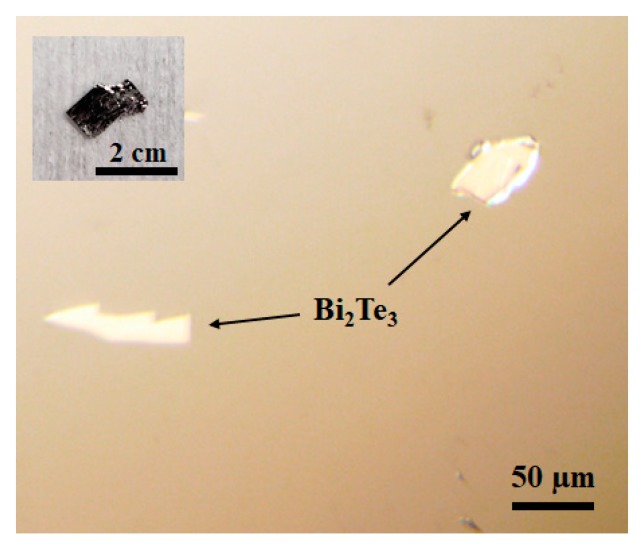
Image of Bi_2_Te_3_ flakes after exfoliation. The inset is showing the Bi_2_Te_3_ crystal before exfoliation.

Bi_2_Te_3_ flakes were mechanically exfoliated onto a Si substrate from a Bi_2_Te_3_ crystal (2D Semiconductors, 99.999% purity). The Si substrate was sequentially cleaned by hydrogen fluoride (HF), acetone, and isopropyl alcohol (IPA) to reduce the surface contamination and oxidation. Images of the Bi_2_Te_3_ crystal before exfoliation and Bi_2_Te_3_ flakes after exfoliation are presented in Figure 1.

### 2.2. Sample Preparation and Characterization

The Bi_2_Te_3_ flake with uniform surface quality was selected and milled by FIB with 30 KeV Ga^+^ ions inside a FEI Nova Nano SEM450 system (Hillsboro, OR, USA). The angle between the ion beam and the electron beam is 52°. After adding the gas-phase Pt, metallic nanostructures were deposited by scanning a 15 KeV electron beam on the selected area. The surface morphology was determined by scanning electron microscope (SEM), and the elementary composition was confirmed by energy-dispersive X-ray spectroscopy (EDX) in the same chamber. 

### 2.3. Cathodoluminescence

**Figure 2:**
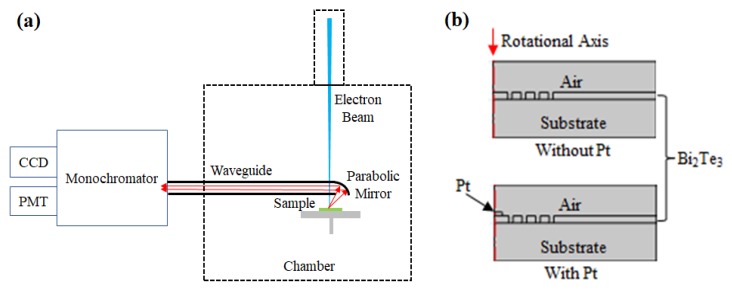
Schematics of (**a**) the cathodoluminescence (CL) setup and (**b**) basic models for the numerical simulation.

The light emission was characterized by cathodoluminescence (CL) in the same system. Since the signal was excited by an electron beam, the beam spot size can be as low as 5 nm. During the CL process, an Al parabolic mirror was inserted above the sample. As indicated in Figure 2a, the focused electron beam passed through a hole on the parabolic mirror and interacted with the sample. The emitted light was collected by the mirror and transmitted by a parallel waveguide to the monochromator. The CL panchromatic images were taken by a photomultiplier tube (PMT) with a functional wavelength from 300 to 900 nm. The wavelength resolved CL spectra were acquired by a CCD camera with a band-pass from 250 to 1000 nm. 

### 2.4. Numerical Simulation

Finally, the electric field distribution at typical wavelengths was simulated by the finite element method using the COMSOL Multiphysics software (version 5.3, COMSOL INC., Burlington, NJ, USA). As indicated in Figure 2b, 2D axisymmetric models were exploited with the rotational axis on the left side. The model used a linear polarized light source with a scattering boundary condition and perfectly matched layers (PMLs). The complex dielectric constants of materials were adapted for the simulation [4,20]. Typical structural parameters were obtained from the experiment, as listed in Table 1.

## 3. Results and Discussion

**Figure 3:**
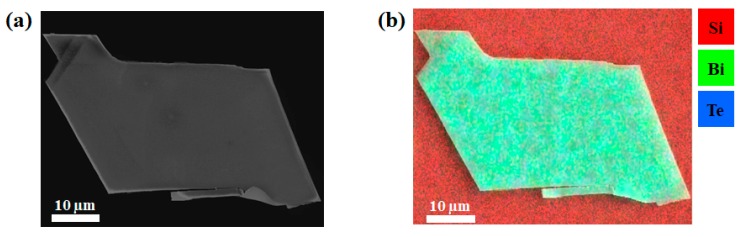
(**a**) SEM image of a Bi_2_Te_3_ flake; (**b**) EDX mapping of the same Bi_2_Te_3_ flake.

**Figure 4:**
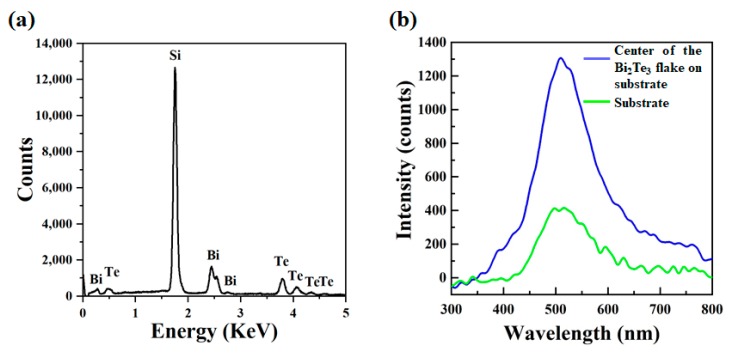
(**a**) EDX spectrum of the same Bi_2_Te_3_ flake; (**b**) CL spectra collected from the Bi_2_Te_3_ flake and the substrate.

The SEM image of a clean and flat Bi_2_Te_3_ flake is presented in Figure 3a. Directly measured in the SEM system, the average thickness of the flake is 190 nm. The EDX mapping of the flake is indicated in Figure 3b. Though the Bi_2_Te_3_ flake has a random shape, the distribution of chemical composition is uniform on the sample, making it a good candidate for further processes. Clear peaks of Bi and Te can be observed in the EDX spectrum, as presented in Figure 4a. The quantified EDX result indicates that the ratio of atomic percentage between Bi and Te is 0.679. As shown in Figure 4b, the blue curve corresponds to the CL spectrum excited at the center of the Bi_2_Te_3_ flake, while the green curve was excited from a spot on the bare substrate far away from the Bi_2_Te_3_ flake. Although peak positions are observed at 510 nm for both curves, the CL intensity on the Bi_2_Te_3_ flake is two times stronger than the substrate. We attribute this effect to the plasmonic enhancement for the semiconductor substrate [21]. Incident electrons first strike the Bi_2_Te_3_ flake and generate surface plasmon waves, then secondary electrons will also interact with the Bi_2_Te_3_ flake when they escape from the surface. Therefore, the surface plasmonic field on the Bi_2_Te_3_ flake will improve the absorption efficiency of the incident beam. Meanwhile, the enhanced electric field across the interface increases the emission efficiency.

**Figure 5:**
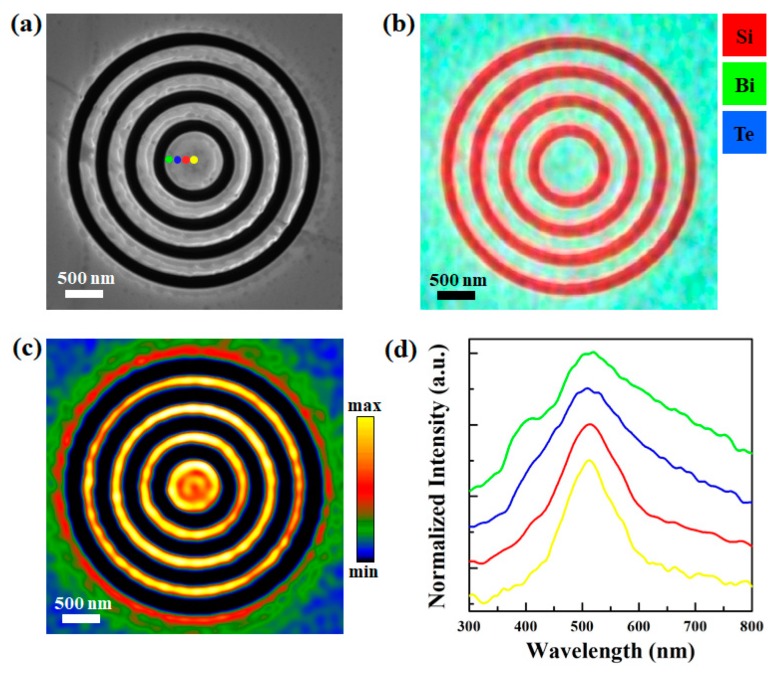
(**a**) SEM image of a bullseye nanostructure on the Bi_2_Te_3_ flake; (**b**,**c**) are the EDX mapping and the CL panchromatic image of the same bullseye nanostructure; (**d**) normalized CL spectra excited at four positions on the central disk.

Bullseye nanoemitters are fabricated on the same flake. As shown in Figure 5a, the SEM image indicates that a Bi_2_Te_3_ bullseye nanostructure with an 800 nm central disk diameter, a 250 nm ring width, and a 125 nm groove width has been made. Although all patterns are milled to reach the Si substrate, no contamination is found inside the gap or on edges, as indicated by the EDX mapping in Figure 5b. The panchromatic CL image of the same bullseye nanostructure is presented in Figure 5c. The CL intensity is obviously higher on the bullseye pattern. The integrated intensity at the geometric center of the Bi_2_Te_3_ bullseye nanostructure is about 1.67 times higher than the integrated intensity on the flake with no pattern. On the central disk, the edge is brighter than the center, indicating that the edge plasmonic modes are dominant. Since electrons are preferred to accumulate at the boundary, the edge behaves similar to a combination of point sources [12]. Surface waves propagate along both positive and negative radial directions [22]. Therefore, the plasmonic resonance increases towards the center and decays outside the pattern. Normalized CL spectra from four locations on the central disk are shown in Figure 5d. These spectra were normalized based on the CL signal excited from the center of the Bi_2_Te_3_ flake. The color of curves corresponds to the color of excitation positions showing as dots in Figure 5a. From the center to the edge, fitted values of the full width at half maximum (FWHM) are 352.69, 236.83, 144.18, and 120.95, respectively. Due to the intense confinement of oscillating charges, stronger plasmonic modes arise at the edge. Therefore, CL emission peaks become wider towards the edge.

**Figure 6:**
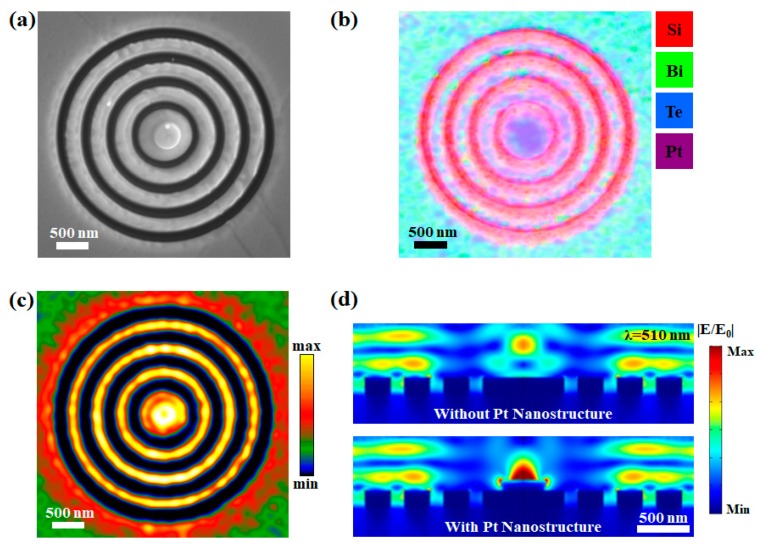
(**a**) SEM image of the Bi_2_Te_3_ nanoemitter with a Pt nanostructure; (**b**,**c**) are the EDX mapping and the CL panchromatic image of the same nanoemitter; (**d**) simulated cross-section electric field distributions of the Bi_2_Te_3_ nanoemitter with and without the Pt nanostructure at 510 nm.

In order to further improve the focusing ability of the Bi_2_Te_3_ bullseye nanoemitter, a cylindrical Pt nanostructure is deposited on the central disk. The SEM image and the EDX mapping have been depicted in Figure 6a,b. The Pt nanostructure has a 400 nm diameter and a 100 nm height. Moreover, the panchromatic CL image after the Pt deposition is presented in Figure 6c. Compared with Figure 5c, the CL emission from the center of the Bi_2_Te_3_ bullseye nanoemitter has been dramatically improved. During the experiment, the incident electron beam first excites the Pt nanostructure. Then, excess electrons continue interacting with the Bi_2_Te_3_ bullseye nanostructure. The emitted light has been better converged due to the plasmonic enhancement of the Pt nanostructure. To explain the mechanism of the field enhancement, cross-section images of the simulated electric field distributions at the peak wavelength (510 nm) are presented in Figure 6d. The simulated mappings are combined with their mirror images to form the whole bullseye nanostructure. For the Bi_2_Te_3_ nanoemitter without the Pt nanostructure, enhanced electric fields are distributed above the surface of the model. Due to the focusing effect of the bullseye structure, a hotspot can be observed above the central disk. When the Pt nanostructure is involved, intense electric fields can be obtained above the Bi_2_Te_3_ grating as well, however, the localized electric field at the geometric center has been dramatically improved. The enhanced electric field on the Pt can be attributed to the coupling effect of the plasmonic emission from the Bi_2_Te_3_ bullseye nanostructure and the localized electric field on the Pt nanodisk [16]. 

**Figure 7:**
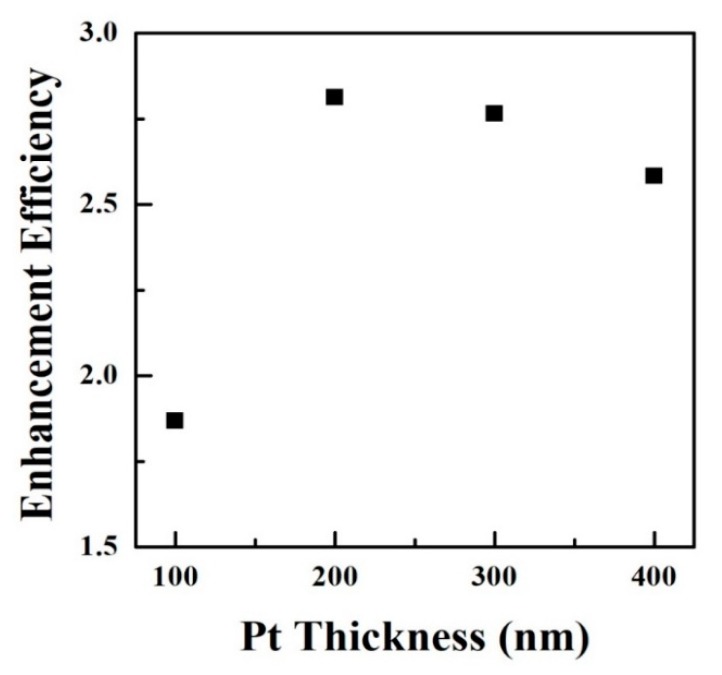
Enhancement efficiencies for nanoemitters with different Pt thickness.

The enhancement efficiency corresponds to the ratio of *I*_1_/*I*_2_, where *I*_1_ and *I*_2_ relate to the integrated CL intensities of the nanoemitter with and without the Pt nanostructure. Figure 7 illustrates the experimental enhancement efficiency as a function of the thickness of the Pt nanostructure. For the same Pt diameter (400 nm), the highest enhancement efficiency is expected to be found on a nanoemitter with the Pt thickness between 200 and 300 nm. The plasmonic emission of the Pt nanostructure increases with the thickness from 100 to 200 nm. On the other hand, due to the shrink of the electron interaction volume inside the sample, the coupling effect attenuates at a higher Pt thickness (>300 nm).

## 4. Conclusions

In this paper, we investigate the plasmonic behavior of Bi_2_Te_3_ bullseye nanoemitters, which are fabricated by FIB milling onto exfoliated Bi_2_Te_3_ nanoflakes. Due to the existence of edge plasmon modes, Bi_2_Te_3_ nanoemitters obtain an excellent focusing property. Excited by the localized electron beam, the CL spectra evolve with the excitation position on the central disk of the bullseye nanostructure. The strongest plasmonic emission can be observed on the edge of the central disk. We propose that the CL intensity at the geometric center can be further improved by adding an extra Pt nanostructure. The numerical simulation indicates that the electric-field enhancement is caused by the coupling effect of the plasmonic emission from the Bi_2_Te_3_ bullseye and the Pt nanodisk. The actual size and geometry of the nanoemitter will be optimized in the future. 

## Figures and Tables

**Table 1:** Typical structural parameters used in the simulation.

Parameter	Description	Value
T1	Thickness of Bi_2_Te_3_ flakes	190 nm
T2	Thickness of the Pt structure	100 nm
R1	Radius of the central disk	400 nm
R2	Radius of the Pt structure	200 nm
W1	Width of grooves	125 nm
W2	Width of rings	250 nm
